# The neuroscience of *Romeo and Juliet*: an fMRI study of acting

**DOI:** 10.1098/rsos.181908

**Published:** 2019-03-13

**Authors:** Steven Brown, Peter Cockett, Ye Yuan

**Affiliations:** 1 Department of Psychology, Neuroscience & Behaviour, McMaster University, 1280 Main St West, Hamilton, Ontario, Canada L8S 4M9; 2 School of the Arts, McMaster University, Hamilton, Ontario, Canada

**Keywords:** acting, fMRI, role playing, theory-of-mind, drama, theatre

## Abstract

The current study represents a first attempt at examining the neural basis of dramatic acting. While all people play multiple roles in daily life—for example, ‘spouse' or ‘employee'—these roles are all facets of the ‘self' and thus of the first-person (1P) perspective. Compared to such everyday role playing, actors are required to portray *other* people and to adopt their gestures, emotions and behaviours. Consequently, actors must think and behave not as themselves but as the characters they are pretending to be. In other words, they have to assume a ‘fictional first-person' (Fic1P) perspective. In this functional MRI study, we sought to identify brain regions preferentially activated when actors adopt a Fic1P perspective during dramatic role playing. In the scanner, university-trained actors responded to a series of hypothetical questions from either their own 1P perspective or from that of Romeo (male participants) or Juliet (female participants) from Shakespeare's drama. Compared to responding as oneself, responding in character produced global reductions in brain activity and, particularly, deactivations in the cortical midline network of the frontal lobe, including the dorsomedial and ventromedial prefrontal cortices. Thus, portraying a character through acting seems to be a deactivation-driven process, perhaps representing a ‘loss of self'.

## 1. Introduction

Dramatic acting is the process of portraying a character in the context of a theatrical performance, where the actor pretends to be someone who s/he is not [1]. However, theatre is not the only context in which role playing occurs in human life. ‘Dramaturgical' approaches to social behaviour argue that social interaction obligatorily involves the playing of situation-specific roles [2,3]. Such roles vary with the context of the interaction and the nature of the interaction partners. The same person can play the role of mother with her child, daughter with her parents, wife with her husband, boss with her employee and customer with a salesperson. While the behaviours associated with these diverse roles may vary strongly, the roles themselves are all facets of the self and thus the first-person (1P) perspective. These variants of the self can be thought of as a series of ‘personas' that are recruited in different social contexts [3]. Many acting teachers, in fact, use this as a starting point for actor training [4,5]. However, playing a character in performance involves a more comprehensive transitioning into a role than playing one of our everyday personas. Actors are required to portray *other* people and to depict their gestures, emotions and behaviours. They are required to live through a set of circumstances and perform a set of actions distinct from their own lives. Actors have to perform on stage not as themselves but as the characters that they are pretending to be [1,6–8]. They thus have to assume what we will call a *fictional first-person* (Fic1P) perspective, in which the ‘I' of their statements applies not to themselves but to the characters who they are portraying at the time.

Acting can be thought of as a form of pretence, in particular the act of pretending to be someone who the actor is not. This idea is central to the acting method derived from the writings of Konstantin Stanislavski [9,10] that dominates the teaching and practice of acting in North America [8,11]. Such pretence shows developmental expression in the form of pretend play in children, which can be thought of as an ontogenetic precursor to theatre [12–16]. Much of the focus of the experimental literature on pretence is on ‘object substitution' [13], for example, how a person can pretend that a banana is a telephone, where such objects become the theatrical props of pretend play [17]. However, much less attention has been devoted to the pretence of *identity* [14] and therefore to the portrayal of characters that permeates both pretend play and theatrical acting. What is being substituted in such situations is not an object, but an identity. The person is no longer behaving as themselves but is pretending to be someone who they are not.

Despite the central importance of role playing to social interaction and despite the ubiquity of pretend play during child development, the topic of role playing has scarcely been examined in experimental psychology or cognitive neuroscience [18], although actors have been studied for other aspects of cognition, like text memorization [19,20]. Instead, there is a large literature devoted to the perceptual phenomenon of theory-of-mind (ToM), which is the process of inferring the intentions, thoughts and emotions of other people [21–24]. ToM can be conceived of as the process of adopting a third-person perspective (3P) on some person (he, she). In many respects, ToM is the perceptual counterpart to role playing as an overt behaviour. It is about *decoding* the intentions of others, whereas role playing is about *displaying* those intentions to people in the context of either everyday role playing or theatrical performances.

A large number of neuroimaging studies have examined the neural basis of 3P perspective-taking (see meta-analyses in [25–31]). For our purposes, the major point of interest is on studies that directly compare 3P and 1P in the same participants; many studies do not do this but instead compare 3P with a non-mentalizing condition. The literature has highlighted the importance of a ‘mentalizing network’ for 3P perspective taking [23,26,28,32–34], comprising the ventromedial prefrontal cortex (vmPFC), posterior cingulate cortex (PCC), precuneus, temporoparietal junction (TPJ), posterior superior temporal sulcus (pSTS) and anterior temporopolar cortex (ATPC). However, almost all of these areas are also activated during self-processing tasks that involve mentalizing [26,34], suggesting that the network is more oriented towards the operation of mentalizing than to ‘other' processing *per se*. While a vast literature of studies has confirmed the importance of this network for mentalizing tasks, what has been far less clear is the *directionality* of the effects between 3P and 1P, with the exception of the TPJ, which generally shows increased activity for 3P compared to 1P in direct comparisons [34–41]. For midline structures like the mPFC and PCC, some studies show relative increases in activation for 3P tasks compared to 1P tasks (e.g. [34–37,42,43]), whereas other studies show relative increases for 1P compared to 3P (e.g. [34,39,44–49]). Hence, while the engagement of these brain areas in mentalizing tasks is not at issue, what is still unresolved is what operations these areas carry out when it comes to perspective taking.

One of the central goals of the present study was to examine the neural basis of acting relative to the known neural network for ToM processing. This is important since there are numerous methods by which actors are able to get into character [1,6–8,50], and some of them are predicated on a mentalistic approach that fully engages perspective taking with the portrayed character. In a general sense, acting methods can be polarized along the lines of being either ‘outside-in' or ‘inside-out' [7,8], although these approaches are thought of by most acting theorists as complementary methods for getting into character. Outside-in approaches are gestural methods that emphasize the physical and expressive techniques of the actor [1,8,50]. Well-known examples in world theatre traditions include Kabuki and No in Japan, Commedia dell'Arte in Renaissance Italy, Peking Opera in China and pantomime traditions in France. Actors focus their training on developing physical skills, often learning highly codified systems of gesturing. The internal or psychological component of the character is thought to emerge out of a mastery of the external forms [51], rather than being the direct emphasis of these methods.

In contrast to this, inside-out approaches are psychological methods that rely on perspective-taking and identification with the character. The dominant form of acting in North America is derived from the theories of Stanislavski [9,10], whose approach is strongly oriented towards interpreting the motivations and emotions of the character and in using this information as a means for identifying with the character. The goal of the method is to produce performances that look natural and that resemble everyday life. Actors appear to be living through the performance as if the events were happening to them. Achieving this can involve a large degree of 3P perspective-taking with the character, such as through studying their personal history in play texts, imagining their circumstances, and deciding on their objectives, obstacles and actions [4,5,52]. However, it is important to keep in mind that, while the process of assuming a 3P perspective on a character may be a central part of the preparatory phrase of learning a role, it should not, according to Stanislavski's method, be an active process during a performance itself [7]. For example, while an actor might develop detailed 3P knowledge about Romeo and his personal situation while studying the play *Romeo and Juliet*, his ultimate goal is to portray Romeo's story in performance as if it were happening to him (Fic1P perspective), not simply to recount details about him (3P perspective) the way that a theatre professor would in lecturing about the character. The commonly understood goal of method acting is for the actor to ‘become' the character in performance.

To the best of our knowledge, only a single neuroimaging study has looked at processes related to assuming a Fic1P perspective, although it was not motivated by a desire to understand acting. Ames *et al*. [47] had non-actor participants perform preference-discrimination tasks either from their own self perspective (1P), from that of an unfamiliar person (3P), or while imagining themselves to be a different, but still unfamiliar, person (Fic1P). For the latter two conditions, participants had to write a narrative essay about the unfamiliar persons prior to scanning, doing so either from the 3P or Fic1P perspective. The study was a region-of-interest analysis focusing exclusively on the vmPFC. The authors found that activity in this region during the Fic1P condition was intermediate in magnitude between that of 1P and 3P, where all three tasks showed deactivations relative to the resting baseline. This creates a prediction for the current study that the vmPFC should be more deactivated during acting than during self processing, although less deactivated than during 3P processing.

The principal objective of the current study was to examine acting (dramatic role playing) for the first time using functional neuroimaging methods. Because of the diversity of acting methods, we wanted to focus on a group of actors having the same general methodological approach towards getting into character. As MRI experiments provide significant constraints on gesturing and facial expression, we opted to use actors with a psychological approach towards getting into character (rather than a gestural one), in particular, the method of Stanislavski. The basic task that the actors performed in the scanner was to answer hypothetical questions (e.g. ‘Would you go to a party you were not invited to?' ‘Would you tell your parents if you fell in love?'), and to do so from different perspectives, including 1P, 3P (as per ToM studies) and Fic1P while portraying either Romeo (male participants) or Juliet (female participants) from Shakespeare's play *Romeo and Juliet*. The 1P task was intended to establish the basic network for mentalizing about the self (e.g. self-awareness). By comparing the Fic1P and 1P conditions, we hoped to identify brain areas involved in acting, i.e. pretending to be some other person. By comparing the Fic1P and 3P conditions, we hoped to determine the extent to which acting takes advantage of the well-characterized brain network for ToM. Simply put, the Fic1P task was intended as a marker of *role* change compared to the self, whereas the 3P task was intended as a marker of *perspective* change compared to the self. Finally, as a means of tapping into gestural acting within the constraints of an MRI experiment, we explored an additional condition in which participants answered questions from their own 1P perspective, but did so using a British accent. Hence, this condition was matched to the 1P condition both for role (i.e. the self) and linguistic content. It was an attempt to examine the gestural aspect of acting independent of a change in identity, where this kind of mimicry is considered as a low-level form of acting [53], one that might be supportive of an ‘outside-in' approach to the development of character. Based on the one prior study that examined role change [47], we predicted that Fic1P would show an intermediate level of vmPFC deactivation compared to the 1P and 3P conditions. Aside from that, it was predicted that there would be relative activation increases in the Fic1P condition in areas involved in character pretence, although the literature did not provide indications as to what such areas might be.

## 2. Methods

### 2.1. Participants

Fifteen actors (11 females, mean age 21 years old, range 18–25 years old; 14 right-handed) participated in the fMRI experiment after giving their informed consent (Hamilton Integrated Research Ethics Board, St Joseph's Healthcare Hamilton). All participants but one were third- or fourth-year Theatre majors at McMaster University. The exception was a person who had previously graduated from the same theatre program and who thus had the same type of acting training, but who was not a student at the time of the study. All of them had performance experience on stage and had studied several Shakespeare plays as part of their coursework. All participants were trained as actors using the psychological ‘method acting' approach to character portrayal. As mentioned in the Introduction, the constraints on movement in an MRI scanner required that we consider participants with a psychological, rather than a gestural, orientation towards getting into character. Participants had normal or corrected-to-normal vision and no history of neurological disorders, psychiatric illness, alcohol or substance abuse, and were not taking psychotropic medications. Participants received monetary compensation for their participation.

### 2.2. Tasks

Participants performed four tasks (one per scan) in a random order. All stimuli consisted of hypothetical questions (Would you do/be…?), to which the participant improvised a response covertly (i.e. without vocalizing aloud). For each trial, a question was presented visually for 6 s, during which time participants were told to reflect on the question but to avoid generating a reply. A dot then replaced the question on the screen, and the participant was told to generate an answer to the question during the next 12 s. They were instructed to elaborate their answers so as to fill the entire 12 s response period. After this period, a cross-hair appeared on the screen for 18 s, and this period of fixation was used as the baseline condition. The eyes were kept open throughout the scanning session. Each task/scan consisted of nine randomly ordered questions and lasted 5 min 24 s (9 questions × 36 s epochs). Questions were displayed using Presentation software (Neurobehavioural Systems, Albany, CA). They were projected visually from an LCD projector onto a screen that was mounted on top of the head coil, with an angled mirror that reflected the light into the participant's field of view.

Slightly different questions were used for each scan, although they all covered identical themes, which included friendship, parents, religion, romance, authority, social status, conflict and death. Examples included ‘Would you go to a party you were not invited to?' (social status), ‘Would you tell your parents if you fell in love?' (romance), and ‘Would you attend the funeral of someone you didn't like?' (death). Different questions were used across conditions in order to avoid priming effects such that recollection of one's response from one perspective would influence processing from another perspective later in the experiment. This was also done to prevent participants from rehearsing Romeo/Juliet answers in situations where the acting scan occurred after one of the other conditions. The questions contained an average of 11.3 words (s.d. = 1.6, range 8–15). Participants were instructed to answer the questions from a different perspective in each scan; no changes of perspective occurred within a scan. Four different perspectives were analysed. (i) *First-person (1P, Self)*: participants answered the questions from their own 1P perspective using the pronoun ‘I'. (ii) *Third-person* (*3P, Theory-of-mind)*: participants answered questions from the perspective of some close ‘other' who they knew well, doing so using the pronoun ‘he' or ‘she'. The questions were placed in the gender format (either he or she) appropriate for the target person. The person was generally a close friend, although a few participants selected romantic partners. The selected person was chosen during a training session on a day prior to scanning. The only criterion for this selection was that the person be of approximately the same age as the participant, thereby excluding a parent or a much younger sibling. In all cases, it was a person who the actor knew very well. There was no constraint on gender; eight of the participants selected a same-gender person (seven of the 11 females and one of the four males). While this selection was uncontrolled, we are not aware of any neuroimaging evidence demonstrating a neural difference between mentalizing about a same-gender versus an opposite-gender person. Compared to typical ToM studies, the questions in our task dealt with the intentions and motivations of people, rather than their beliefs and emotions. They dealt with predictions of behaviour, secondary to thoughts and feelings. They relate to what Nichols & Stich [22] refer to as the ‘planner' component of 3P perspective-taking in which the person attempts to ascertain the goals and behavioural plans of a person in a given situation. (iii) *British accent*: participants were instructed to answer the questions from their own 1P perspective using the pronoun ‘I' (exactly as in the 1P condition), but to do so using a British accent. Participants were explicitly told not to change their identity to that of some British person, but to answer the questions with exactly the same content as they themselves would. We confirmed during a training session that the participants performed the task properly by comparing their answers with similar practice questions posed in the 1P condition. Casual observation of the participants performing the task vocally during the training session confirmed that all of the participants were able to create a convincing British accent with little effort. No participant complained about having difficulties performing the task. (iv) *Fictional first-person* (*Fic1P)*: participants were told to get into the character of Romeo (male participants) or Juliet (female participants) and to answer the questions from the 1P perspective of their character using the pronoun ‘I’. This was situated in terms of the thoughts, beliefs and emotions of the characters at the time of the balcony scene (Act 2, Scene 2) of Shakespeare's *Romeo and Juliet*, which occurs shortly after the lovers' first encounter in the play. Identical questions were used for male/Romeo and female/Juliet participants.

Participants were given as much time as they needed before the acting scan in order to get into character, for example, by reciting lines from the play or imagining the emotions and motivations experienced by their character during the balcony scene of *Romeo and Juliet*. All of the participants had memorized their character's lines for the balcony scene. Participants in the scanner took between 10 and 120 s to do this, with the mean time being 67 s (SD = 35 s). They signalled their readiness to start the scan by squeezing the in-scanner alarm bell. Participants were told that, since the stimuli were hypothetical questions, rather than factual questions, there were no right or wrong answers to these questions and that the most important facet of the task was to feel in character, rather than to formulate ‘correct' answers. During the training session (see below), participants were reminded of the fact that they themselves possessed third-person knowledge of these characters based on their study of the play, but that they should answer the questions the way that the *character* would, not the way they themselves would. During the baseline condition, participants fixated on a cross-hair. For the baseline epochs during the acting condition, the actors were explicitly told not to do any type of rehearsal, but to simply relax while staying in character. After the scanning session was over, the participants were asked to rate on a 10-point scale the extent to which they felt that they were in character during the Fic1P scan, where one indicated not at all and 10 indicated completely. The mean rating was 7.5 (s.d. = 0.8), where the values ranged from 6 to 9.

It is important to note that an alternative manner of having participants do an acting task would have been to recite scripted passages from well-known plays. For example, the participants could have recited monologues from *Romeo and Juliet* while in character. This would have been a more naturalistic approach to acting. However, we opted against the scripted approach because we wanted to place acting in opposition to both self processing and ToM, and so we wanted a task that could generalize across these perspectives. Composing and memorizing scripted monologues from the 1P and 3P would have been very unnatural and would not have tapped into cognitive processes of perspective taking. Therefore, we decided on an improvisational task that required the participants to generate novel responses for each perspective. One could criticize our Fic1P task by saying that participants were devoting more resources to determining what Romeo or Juliet would say in a given situation than in true role playing, as they would have to tap into their 3P knowledge of the play and their understanding of the characters. But one could make the same critique of the 3P perspective, since ToM too is an act of forming inferences about another person. In the realm of actor training, a question-and-answer format is a common technique for character-building in ‘hot-seating', in which actors are placed in a chair and asked to answer questions while in character. This technique is used because the pressure to improvise answers in the moment can result in a stronger connection with one's character than simply reflecting on the character *per se*. What is significant for our Fic1P task is that it required a *role change* and the use of the pronoun ‘I', not ‘he' or ‘she', a shift that is central to training in the method system of acting [5,52,54].

### 2.3. Training sessions

All participants underwent two types of training sessions on a day prior to scanning. First, each participant took part in a 1 h training session with the first and last authors in order to become familiar with the nature of the tasks and the timing of the trial structure, but not the questions to be used in the scanner (practice questions were used instead). During this session, participants were asked to answer the questions aloud so that we could ensure that they understood the timing of the trial. In the scanner, by contrast, participants responded covertly to all questions. Second, participants underwent 1–3 h of ‘workshop' training with the second author (an actor, acting professor and theatre director) oriented towards Shakespeare's *Romeo and Juliet*. This was generally done in small groups. These sessions began with the rehearsal of a monologue from *Romeo and Juliet*'s balcony scene, as informed by a conventional rehearsal-discussion of the characters' backgrounds, such as their social and cultural contexts, their objectives, and their obstacles. The sessions finished by ‘hot-seating' the participants, which involved challenging them to improvise answers to questions in character in the first person by adopting the perspective of the character they were portraying. At no time were the actual questions from the MRI experiment ever used in these workshop sessions. In fact, the second author was blind to the questions used in the experiment. Participants did not encounter the actual questions until they were scanned.

### 2.4. Image acquisition

Magnetic resonance images were acquired with a GE Medical Systems Signa Excite 3-Tesla MRI at the Imaging Research Centre at St Joseph's Healthcare Hamilton. Functional images sensitive to the blood-oxygen-level-dependent (BOLD) signal were collected with a gradient-echo pulse sequence using standard parameters (TR = 2000 ms, TE = 45 ms, flip angle = 90°, 31 slices, 4 mm slice thickness, no gap, matrix size = 64 × 64, field of view = 24 cm, voxel size = 3.75 × 3.75 × 4 mm), effectively covering the whole brain. All functional scans lasted 5 min 24 s, resulting in the collection of 162 brain volumes per scan. Four dummy scans were run before data acquisition began. High-resolution, T1-weighted structural images were acquired in order to register functional activity onto brain anatomy. The scanning parameters were 3D-FSPGR, IR-prepped, Ti = 450 ms, flip angle = 12 degrees, TR = 7.5 ms, TE = 2.1 ms, field of view = 240 × 180 mm, slice thickness = 2 mm, acquisition matrix 320 × 192, 1 average, receiver bandwidth = 31.25 kHz, data were interpolated to 512 × 512 matrices, and number of slices doubled during reconstruction, resulting in 154 slices.

### 2.5. Data analysis

The data were analysed using a slow event-related design. Functional images were reconstructed offline, and the scan series was realigned and motion-corrected using BrainVoyager QX 2.8. Motion-correction analysis revealed that participants displayed very little head movement, as expected for a covert task. Translational and rotational corrections did not exceed an acceptable level of 1 mm and 1 degree, respectively, for any participant. During the preprocessing stage, a temporal high-pass filter was applied at a frequency of 0.0078 Hz, or 2 cycles per scan, using the GLM-Fourier algorithm. Three-dimensional spatial smoothing was performed using a Gaussian filter with a full-width-at-half-maximum kernel size of 4 mm. Following realignment, each functional scan was normalized to the Talairach template [55]. The BOLD response for each task-block was modelled as the convolution of an 18 s boxcar with a synthetic haemodynamic response function composed of two gamma functions, reflecting the contrast between task epochs and fixation. Beta weights associated with the modelled haemodynamic responses were computed to fit the observed BOLD-signal time course in each voxel for each participant using the general linear model, as implemented in Brain Voyager QX. The six head-motion parameters were included as nuisance regressors in the analysis.

**Figure 1.**
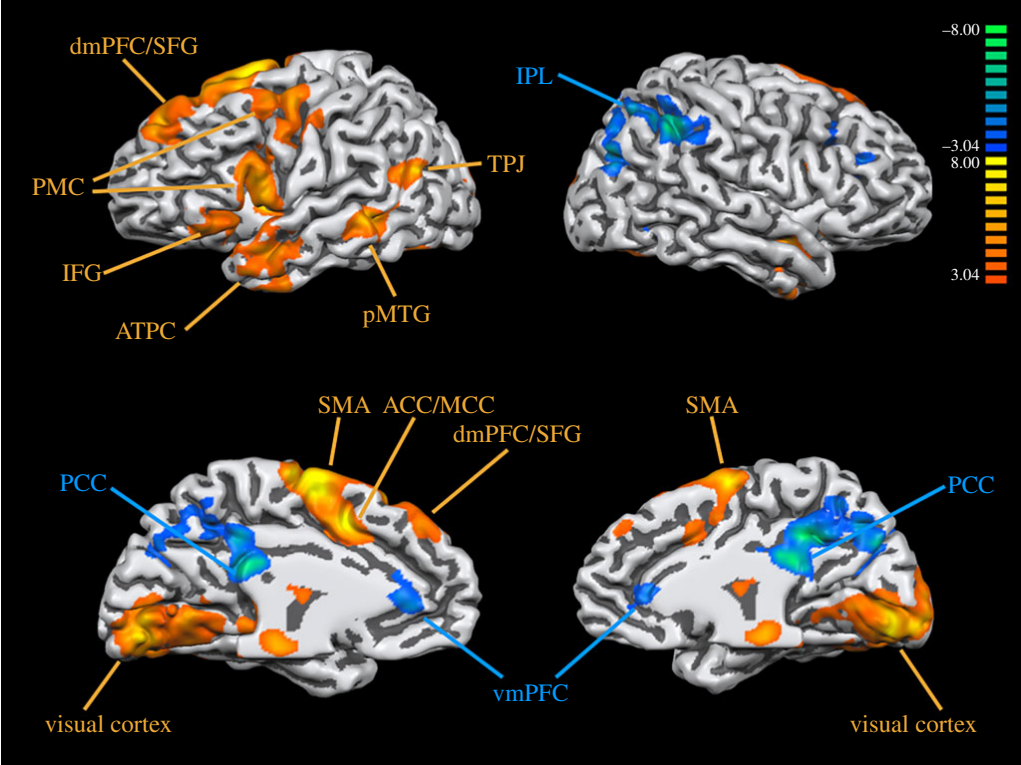
Brain activations and deactivations for the first-person (1P) task compared with fixation, registered onto an inflated template brain. The results shown are FDR corrected *p* < 0.05. Abbreviations: ACC/MCC, anterior cingulate cortex/midcingulate cortex; ATPC, anterior temporopolar cortex; dmPFC/SFG, dorsomedial prefrontal cortex/superior frontal gyrus; IFG, inferior frontal gyrus; PCC, posterior cingulate cortex; PMC, premotor cortex; pMTG, posterior middle temporal gyrus; SMA, supplementary motor area; TPJ, temporoparietal junction; vmPFC, ventromedial prefrontal cortex. Activations are labelled orange, and deactivations are labelled blue.

**Figure 2.**
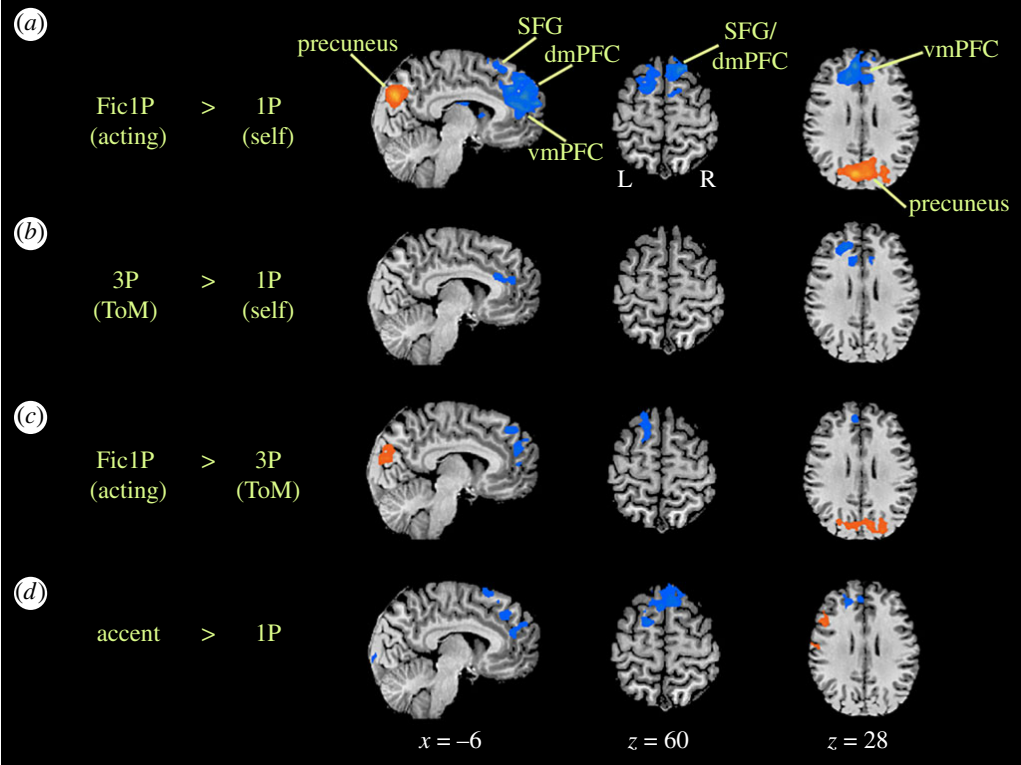
High-level contrasts. Results are registered on the anatomical MRI of one of the participants (P9). (*a*) Acting (Fic1P) versus self (1P), (*b*) theory-of-mind (3P) versus self (1P), (*c*) acting (Fic1P) versus theory-of-mind (3P) and (*d*) British accent versus self (1P). These contrasts were thresholded with cluster-level correction for multiple comparisons to control for the rate of false positives. An initial cluster-forming threshold of *p* < 0.05 uncorrected was applied, followed by a Monte Carlo simulation using the AlphaSim algorithm in Brain Voyager, which selected a cluster-size threshold for each high-level contrast that maintained a family-wise error rate of *p* < 0.05. The obtained *k*-value (in voxels) was 100 for Fic1P versus 1P, 82 for 3P versus 1P, 90 for Fic1P versus 3P, 93 for accent versus 1P and 95 for Fic1P versus accent. dmPFC, dorsomedial prefrontal cortex; PCC, posterior cingulate cortex; SFG, superior frontal gyrus: vmPFC, ventromedial prefrontal cortex.

Each participant's data were processed using a fixed-effects analysis, where a false discovery rate (FDR) of *p* < 0.05 was employed as a correction for multiple comparisons. Contrast images for each participant were brought forward into a random-effects analysis with FDR correction at *p* < 0.05. The group data shown in figure 1 were registered onto an inflated brain of one of the participants within the study (P9), as generated using Brain Voyager. High-level contrasts were thresholded with cluster-level correction for multiple comparisons to control for the rate of false positives. An initial cluster-forming threshold of *p* < 0.05 uncorrected was applied, followed by a Monte Carlo simulation using the AlphaSim algorithm in Brain Voyager, which selected a cluster-size threshold for each high-level contrast that maintained a family-wise error rate of *p* < 0.05. The obtained *k*-value (in voxels) was 100 for Fic1P versus 1P, 82 for 3P versus 1P, 90 for Fic1P versus 3P, 93 for accent versus 1P and 95 for Fic1P versus accent. The results are reported as *t*-tests. Deactivations are defined in all cases as the reverse contrast. However, in order to keep the nomenclature of the contrasts consistent, we have maintained the same contrast names throughout the Results section, but reported activations (i.e. positive *t*-values) in table 2 and deactivations (i.e. negative *t*-values) in table 3. For the Fic1P versus 1P contrast, for example, describing deactivations in this way is equivalent to reporting positive *t*-values for the reverse 1P versus Fic1P contrast. Because figure 2 shows activations and deactivations on the same slices, we prefer to keep the contrast names consistent for the activations and deactivations, and instead reverse the sign of the *t*-values in the respective tables for activation (table 2) and deactivation (table 3).

## 3. Results

Figure 1 shows activations and deactivations for the 1P condition (self processing) against fixation, with Talairach coordinates for the activations for all four tasks listed in table 1. (Versions of the coordinate tables that contain cluster sizes are found in the electronic supplementary material). This is done in order to verify that all of the tasks activated expected sensorimotor brain areas for the kind of question-answering task performed in this study; acting-specific activations are discussed below. The 1P versus fixation subtraction highlights the basic sensorimotor activations found in all of the conditions in the study. Activity was seen in visual areas such as V1 and V2 involved in reading the stimulus questions. Activations involved in the subvocal generation of answers included premotor areas related to vocalization (left vocal premotor cortex, supplementary motor area (SMA), cingulate gyrus and bilateral cerebellum) and those related to lexical selection and language generativity (left middle temporal gyrus, left inferior frontal gyrus). Left-hemisphere activations were seen in the superior frontal gyrus (SFG). Strong bilateral activations in the caudate nucleus were most probably coupled with the prefrontal activations. Mentalizing-related activations were seen in the ATPC bilaterally and the left TPJ. Deactivations were seen bilaterally in the region of the PCC and the vmPFC, in keeping with the known activity of these areas in the resting state network [56–58]. Additional deactivations were seen in the inferior parietal lobule of the right hemisphere.

**Table 1.** Talairach coordinates of the activations for the four tasks versus fixation. The results are FDR corrected *p* < 0.05. After each anatomical name is the Brodmann area (BA) for that region in parentheses. The columns labelled as *x*, *y* and z contain the Talairach coordinates for the peak of each cluster reaching significance. The *t*-value is the maximal value for that cluster. In order to keep the table a manageable size, only activations of *t* > 6 are shown. ATPC, anterior temporopolar cortex; dmPFC, dorsomedial prefrontal cortex; TPJ, temporoparietal junction; SMA, supplementary motor area; STG, superior temporal gyrus.

	**1P**				**3P**				**Fic1P**				**accent**			
	*x*	*y*	*z*	*t*	*x*	*y*	*z*	*t*	*x*	*y*	*z*	*t*	*x*	*y*	*z*	*t*
left hemisphere																
*frontal lobe*																
SMA (BA 6)	−6	−1	61	12.5	−6	−1	64	11.3	−3	−4	64	11.1	−6	−1	58	10.5
	−3	−10	67	10.0	−6	−10	67	8.4					−3	−10	67	9.2
superior frontal gyrus (BA 6)	−12	20	58	7.3												
cingulate gyrus (BA 32)	−6	5	46	9.7	−9	8	43	12.1					−9	5	43	10.2
inferior frontal gyrus (BA 44)	−45	5	13	7.0					−54	8	16	7.8	−45	5	13	8.3
									−57	17	13	8.8	−57	8	16	7.9
inferior frontal gyrus (BA 45)					−57	14	22	8.6								
dmPFC (BA 8)					−12	44	46	8.3								
middle frontal gyrus (BA 6)					−39	2	61	7.0								
*temporal lobe*																
ATPC (BA 21/38)	−42	2	−35	8.3					−48	−1	−35	9.0	−48	−1	−35	7.5
									−48	17	−23	7.6	−51	8	−11	7.7
TPJ (BA 39)	−48	−64	22	7.7												
middle temporal gyrus (BA 21)	−60	−37	1	7.5	−54	−43	1	7.2	−54	−37	4	6.9	−51	−43	4	8.2
anterior STG (BA 22)													−54	−1	−5	7.4
*occipital lobe*																
lingual gyrus (BA 18)	−12	−79	−2	7.6	−3	−82	−5	11.8								
*subcortical*																
putamen	−27	−1	4	15.4	−21	2	10	11.3					−21	−4	4	9.0
substantia nigra	−9	−19	−8	8.6									−15	−22	−8	10.0
thalamus					−15	−16	7	7.0					−12	−16	10	8.4
globus pallidus					−18	−7	−2	8.5	−18	−10	4	7.0				
caudate nucleus													−18	−16	25	10.0
													−18	8	16	9.9
*cerebellum*																
lobule VI	−36	−73	−23	7.3												
lobule V	−12	−58	−20	7.2												
lobule IV													0	−49	−8	7.5
right hemisphere																
*frontal lobe*																
SMA (BA 6)	6	−4	61	9.2												
insula (BA 13)													39	20	4	7.5
*temporal lobe*																
anterior STG (BA 38)	54	2	−8	7.3					48	5	−11	7.0	51	2	−8	8.2
*occipital lobe*																
lingual gyrus (BA 18)	3	−79	−8	8.9	9	−79	−8	7.9	9	−79	−2	9.1	9	−76	−2	8.2
*subcortical*																
caudate nucleus	15	2	19	16.0	18	2	19	9.9					15	11	13	10.1
putamen	18	5	10	11.0	18	−4	4	7.1					15	−4	4	9.6
*cerebellum*																
lobule VI	30	−52	−29	10.5	30	−55	−26	7.4	39	−55	−32	7.7	18	−67	−26	9.3
lobule V													12	−58	−20	10.2
crus I	27	−79	−29	8.3	27	−73	−32	8.1					30	−73	−29	7.3

Figure 2 shows the results of the four high-level contrasts analysed in the study, with Talairach coordinates presented in table 2 (activations) and table 3 (deactivations). Figure 2*b* shows the ToM contrast, comparing 3P with 1P. The goal of carrying out this contrast was to perform a sanity check with regard to the literature on perspective taking, as well as to establish a set of brain areas for mentalizing on its own that could be used as a point of reference when examining the brain areas for acting in the Fic1P versus 1P contrast (below). The activation pattern was quite similar to that of 1P, reflecting the overall similar sensorimotor nature of the question-answering task. The contrast showed a deactivation in the vmPFC and the ventral part of the dmPFC (dmFPC-v). Another mentalizing area that showed deactivation in this contrast was the left ATPC (BA 38; table 3). There were no areas that were more active in 3P than 1P, only deactivations. Hence, coordinates for the 3P versus 1P contrast are only found in table 3 (deactivations), and not table 2 (activations).

**Table 2.** Talairach coordinates of the activations for four of the high-level contrasts. The contrasts were thresholded with cluster-level correction for multiple comparisons to control for the rate of false positives. An initial cluster-forming threshold of *p* < 0.05 uncorrected was applied, followed by a Monte Carlo simulation using the AlphaSim algorithm in Brain Voyager, which selected a cluster-size threshold for each high-level contrast that maintained a family-wise error rate of *p* < 0.05. The obtained *k*-value (in voxels) was 100 for Fic1P versus 1P, 82 for 3P versus 1P, 90 for Fic1P versus 3P, 93 for accent versus 1P and 95 for Fic1P versus accent. 3P versus 1P did not show any activations; its deactivations are shown in table 3. After each anatomical name is the Brodmann area (BA) for that region in parentheses. The columns labelled as *x*, *y* and *z* contain the Talairach coordinates for the peak of each cluster reaching significance. The *t*-value is the maximal value for that cluster. DLPFC, dorsolateral prefrontal cortex; ITG, inferior temporal gyrus; MOG, middle occipital gyrus; MTG, middle temporal gyrus; PMC, premotor cortex; pSTS, posterior superior temporal sulcus; TPJ, temporoparietal junction.

	Fic1P > 1P				Fic1P > 3P				accent > 1P				Fic1p > accent			
	*x*	*y*	*z*	*t*	*x*	*y*	*z*	*t*	*x*	*y*	*z*	*t*	*x*	*y*	*z*	*t*
precuneus (BA 31/19)	−6	−73	28	6.8	18	−79	34	3.5					6	−73	28	4.1
	9	−76	31	5.1	−24	−70	28	3.2					15	−70	22	3.3
					24	−79	22	3.5								
precuneus (BA 7)	9	−82	40	4.1												
dorsal precuneus (BA 7)	18	−79	55	4.2												
TPJ (BA 39)	54	−64	16	4.1									42	−61	22	3.4
pSTS (BA 22/21)	39	−55	7	3.3	51	−58	10	3.6								
MTG (BA 37)					−48	−67	4	3.3								
					42	−61	4	3.5								
fusiform gyrus (BA 19)					−33	−67	−11	4.0					−45	−91	−11	6.1
cuneus (BA 18)					18	−70	19	3.9								
MOG (BA 37)					24	−52	−8	3.0					27	−49	−8	3.7
MTG (BA 39/19)													39	−73	13	4.3
													−18	−79	13	3.0
orofacial PMC (BA 6)									−54	−7	40	4.1				
DLPFC (BA 9)									−48	29	37	4.0

**Table 3.** Talairach coordinates of the deactivations for the five high-level contrasts. The contrasts were thresholded with cluster-level correction for multiple comparisons to control for the rate of false positives. An initial cluster-forming threshold of *p* < 0.05 uncorrected was applied, followed by a Monte Carlo simulation using the AlphaSim algorithm in Brain Voyager, which selected a cluster-size threshold for each high-level contrast that maintained a family-wise error rate of *p* < 0.05. The obtained *k*-value (in voxels) was 100 for Fic1P versus 1P, 82 for 3P versus 1P, 90 for Fic1P versus 3P, 93 for accent versus 1P and 95 for Fic1P versus accent. After each anatomical name is the Brodmann area (BA) for that region in parentheses. The columns labelled as *x*, *y* and *z* contain the Talairach coordinates for the peak of each cluster reaching significance. The *t*-value is the maximal value for that cluster. In order to keep the table a manageable size, only deactivations of *t*<−3 are shown. ATPC, anterior temporopolar cortex; dmPFC-d, dorsal part of the dorsomedial prefrontal cortex; dmPFC-v, ventral part of the dorsomedial prefrontal cortex; IFG-PO, inferior frontal gyrus pars orbitalis; MOG, middle occipital gyrus; PHG, parahippocampal gyrus; SFG-ant, anterior part of the superior frontal gyrus; SFG-post, posterior part of the superior frontal gyrus; SMA, supplementary motor area; STG, superior temporal gyrus; vmPFC, ventromedial prefrontal cortex.

	Fic1P > 1P				3P > 1P				Fic1P > 3P				accent > 1P				Fic1p > accent			
	*x*	*y*	*z*	*t*	*x*	*y*	*z*	*t*	*x*	*y*	*z*	*t*	*x*	*y*	*z*	*t*	*x*	*y*	*z*	*t*
SFG-post (BA 6)	9	8	58	−4.6									−15	5	55	−6.1				
	−12	8	61	−3.6																
SFG-ant (BA 6)	−12	29	55	−4.2					−15	29	58	−4.0								
	15	26	61	−4.4					−12	20	61	−3.1								
dmPFC-d (BA 8)	−12	50	46	−4.1					−9	41	46	−4.5	15	44	55	−4.3				
									−21	44	49	−3.0	18	35	49	−4.2				
dmPFC-v (BA 8/9)	−3	38	37	−4.4																
	−21	41	34	−4.1	−18	44	34	−4.6												
	12	32	37	−3.8	−30	38	31	−3.7												
	9	50	34	−3.5					0	56	37	−3.0	0	50	34	−3.9				
vmPFC-d (BA 9)	−6	50	28	−5.5	−12	20	28	−3.5	−6	50	28	−3.5	−18	47	25	−3.6	−15	38	28	−4.8
	3	53	19	−5.4	12	23	25	−3.7												
	−3	44	19	−5.3																
vmPFC-v (BA 10)	−12	47	10	−3.3					−9	50	10	−4.1	6	56	10	−4.2				
SMA (BA 6)																	0	−7	61	−4.9
insula					−36	−7	−2	−3.5												
STG (BA 22)					−45	−28	1	−4.4												
IFG-PO (BA 47)					−30	41	−8	−4.5												
					−39	23	−8	−4.1												
PHG (BA 36)					−27	−37	−8	−3.2												
ATPC (BA 38)					−45	14	−35	−3.1												
MOG (BA 19)													33	−79	13	−3.6				
													−18	−79	13	−3.6				
MOG (BA 18)													−33	−88	1	−5.0				
caudate nucleus	6	2	19	−5.1													−21	−4	16	−5.3
	−12	8	13	−4.2													−18	5	10	−4.4
putamen																	15	8	7	−5.3
cerebellum (crus I)													27	−79	−29	−5.1				

Figure 2*a* shows the contrast of interest for the study, namely acting (Fic1P) compared to self-processing (1P). Deactivations were seen in anterior parts of the cortical midline. This included the same part of the vmPFC and dmPFC-v that was seen in the 3P versus 1P contrast. However, it also included a large part of dmPFC/SFG that was not seen in the ToM contrast. Bilateral deactivations were seen in the caudate nucleus, most probably associated with those in the medial PFC. Activations were only seen in the precuneus and the region of the pSTS and TPJ (table 2). The latter is a region commonly seen in ToM tasks, although it did not appear in the 3P versus 1P contrast in this study, and was specific to the acting contrast.

Figure 2*c* shows the contrast between acting and the 3P condition in order to examine the extent to which acting depends on activation changes that occur during ToM tasks. The Fic1P-related deactivation in the dmPFC/SFG persisted in the Fic1P versus 3P contrast, although only in the left hemisphere. A weak deactivation in the vmPFC was present in this contrast as well. The precuneus activation seen in the Fic1P versus 1P contrast was also present in Fic1P versus 3P, as well as a weak activation in the right pSTS and several occipital areas.

Figure 2*d* shows the British accent condition compared to self processing using one's regular manner of speaking (1P). This contrast controlled for both the self perspective and the content of the answers (which were semantically similar to those for 1P), but introduced a type of gestural modification that could be seen as being a form of pretence or mimicry, even though the participants were explicitly told not to imagine that they were a British person, but to answer as themselves. To our surprise, this accent mimicry led to a similar deactivation pattern as the contrast of acting (Fic1P) with 1P, with deactivations seen in both the dmPFC/SFG and vmPFC. However, the acting-specific activations in the precuneus and TPJ/pSTS were not present, making these activations important markers of the acting manipulation. In support of this, the precuneus activation was present in the contrast of Fic1P versus British accent (table 2). The results of the British accent condition suggest that gestural change can produce a departure from the self along the same lines as acting, although in a weaker manner. Interestingly, a major region of activation increase for British accent compared to 1P was in the vocal premotor cortex [59] of the left hemisphere, perhaps suggestive of the phonological resources required for producing a foreign accent, an effect not seen in the Fic1P versus 1P contrast.

## 4. Discussion

Despite the central importance of role playing in both everyday social interactions and the theatrical arts, it has been little studied in either the psychology or neuroscience literatures. We sought to address this neglect by carrying out the first neuroimaging study of dramatic role playing, employing trained actors as participants. Because of the wide diversity of actor-training methods, we opted to work with a uniform population of actors, all of whom had a similar training in the dominant form of acting in North America and who possessed a similar amount of training and performance experience. In addition, because MRI experiments are so restrictive of body movement and facial gesturing, we chose to examine a group of actors specialized in the mentalistic approach to character portrayal most commonly taught in theatre schools.

The imaging results showed that acting led to deactivations in brain areas involved in self processing, with a focus on the dmPFC/SFG and vmPFC. This might suggest that acting, as neurocognitive phenomenon, is a suppression of self processing. The major increase in activation associated with role change was seen in the posterior part of the precuneus. Perhaps the most surprising finding of the study was that the British accent condition—during which the participants were instructed to maintain their self-identity—showed a similar deactivation pattern vis-à-vis the self that acting did, suggesting that gestural mimicry of even a completely unspecified other has an impact on brain areas involved in self processing. This supports the contention of acting theorists that gestural and psychological approaches might be related paths towards achieving the same goal, namely the embodied portrayal of a character [8,51,60]. It also lends support to theories of embodied cognition, which argue that a change in gestural expression can influence the way that people think and the emotions that they feel [61,62].

We are aware of only a single prior study that attempted anything like our Fic1P condition, that of Ames *et al*. [47], although their task was not intended as an acting condition *per se*. While our vmPFC deactivations were quite a bit dorsal to theirs, we found a similar increase in deactivation for Fic1P compared to 1P, as well as for 3P compared to 1P. The British accent showed a comparable deactivation in the vmPFC to the 3P condition (when both were compared with 1P), but less than that for acting. However, the most acting-specific deactivation we observed was not in the vmPFC but in the dmPFC/SFG. In addition, we observed an area of activation increase in the precuneus that was specific to acting compared to the other three conditions, although it emerged through the loss of deactivation compared to 1P. We would like to consider these findings in terms of the two processes of perspective change and role change.

### 4.1. Perspective change

One of the major objectives of the study was to compare the pattern of brain activation for acting with that for the well-studied process of 3P perspective taking, referred to as ToM processing. In several respects, our study was biased towards seeing an overlap between acting and mentalizing. First, the participant population consisted of actors with a mentalistic orientation towards getting into character that emphasizes inferring the thoughts, emotions and motivations of a character in a given situation. Second, our scanner task required participants to engage in the psychological process of formulating responses to hypothetical questions. It is perhaps not surprising that answering questions as Romeo or Juliet would tap not only into role playing but mentalizing as well in order for the participant to determine an appropriate answer from the perspective of the character. In other words, an actor would have to consult some degree of third-person knowledge about Romeo or Juliet—just as with *any* person other than the self—in order to formulate answers from their perspective. So, neural similarities between Fic1P and 3P may be more of a reflection of our question-answering task than of the nature of acting, since acting theorists believe that mentalizing about a character is far more important during the preparatory phase of studying a role than during the process of character portrayal in performance [7].

With respect to our ToM contrast, the most significant difference between 3P and 1P was a deactivation in the vmPFC and the ventral part of the dmPFC, which became the major marker of perspective change in this study. This same deactivation was seen more intensely in the contrast of acting to 1P. In both cases, the effect resulted from a decrease in the level of activation compared to the self condition. Hence, the presence of the vmPFC deactivation for acting might indicate that actors engage in 3P perspective-taking with their character while undergoing the process of acting, or that acting is a more intense form of perspective taking, since there was a greater reduction in vmPFC and dmPFC-v activities for Fic1P than for 3P. This interpretation is only reasonable if this process occurs in an *implicit* manner. This would also account for the same, but weaker, deactivation effect seen in the British accent condition. While participants in the 3P condition were told to explicitly assume the perspective of their close other, this was not the case during either the acting or accent conditions. If anything, during the accent condition, participants were clearly instructed not to stray from the self perspective. Hence, if an increasing deactivation in the vmPFC and dmPFC-v is a marker of deviation from the self perspective, then this would have to work during both explicit tasks (like 3P and other types of ToM tasks) and implicit tasks where people psychologically stray from the self perspective, but in which they are not told to assume another person's perspective. Van Overwalle & Vandekerckhove [63] reviewed both electroencephalography and fMRI evidence suggesting that implicit mentalizing employs the same mentalizing network as explicit mentalizing. If anything, the medial PFC has been shown to be more strongly linked with implicit than explicit mentalizing [64]. Similar results were obtained when comparing implicit and explicit forms of trait judgement [65].

### 4.2. Role change

Acting produced additional effects beyond the ones in the vmPFC and ventral dmPFC that were observed in the 3P condition, suggesting that acting is something more than just mentalizing about a character. One of these effects was a deactivation in the dmPFC/SFG for acting versus self that was also observed in British accent versus self, while the other was an activation increase in the precuneus that was found when acting was contrasted with either self (1P) or other (3P).

*dmPFC and SFG*. A large dorsoventral extent of the dmPFC was shown to be deactivated during acting when compared to the 1P condition, compatible with *the loss of a self-related process* during acting and gestural pretence. Denny *et al*.'s [26] meta-analysis of self/other processing argued for a dorsoventral distinction in the medial PFC such that the vmPFC showed an overlap between self and other processing (when each one was compared against a low-level baseline), while the dmPFC showed a preference for other compared to self processing. Our results seem incompatible with those findings, since we observed the highest level of dmPFC activity in the self condition, and less in each of the other conditions. Therefore, we would like to consider another dimension of self processing that may be tracked in our results.

D'Argembeau *et al*. [48] carried out a study of trait judgements about the self, but did so across the mental time frames of ‘present self' and ‘past self', as well as ‘present other' and ‘past other'. A peak in the dmPFC at MNI coordinate −2, 56, 26 (compared to Talairach coordinate −6, 50, 28 in Fic1P versus 1P) showed greater activity for the present self than the other three conditions. In addition, another dmPFC peak at MNI coordinate 4, 46, 44 (compared to Talairach coordinate −3, 38, 37 in Fic1P versus 1P) showed an interaction effect such that it was more active for the present self than the other three conditions. D'Argembeau *et al*. [66] followed up on these findings and showed that the dmPFC's preference for the present self extends beyond the past self to include the future self as well. Van der Cruyssen *et al*. [67] showed that both of the regions just mentioned were more active when people processed information about social categories of people (e.g. ambulance driver, kindergarten teacher) than simply adjectival trait descriptions (e.g. attentive, picky). Consistent with these studies on trait judgements about the self and others, the activation likelihood estimation (ALE) meta-analysis of Schurz *et al*. [28] showed that a dmPFC peak at Talairach coordinate 6, 26, 55 (compared to Talairach coordinate −12, 29, 55 in Fic1P versus 1P) is more active when people perform trait judgements than when they do false-belief mentalizing tasks. Likewise, Benoit *et al*. [68] found that the more ventral parts of the dmPFC have a preference for self over other trait-judgements, and Ma *et al*. [65] showed that the more dorsal part of the dmPFC is more active during spontaneous compared to intentional trait judgements. Garrison *et al*. [69] carried out a study in which participants were asked to make judgements about adjectives in terms of the self (Does the word describe you? Yes or no). The results of this task showed strong activations throughout the dorsoventral extent of the dmPFC. By contrast, several parts of the dmPFC were deactivated (when contrasted with rest) during a mindfulness meditation condition in the same participants, perhaps suggesting a reduction in the embodied self through meditation. Therefore, the dmPFC might encode more-enduring and stable features of the self, rather than a person's current mental states, the latter of which are examined in ToM tasks. If so, then the deactivations seen in the dmPFC for acting would represent a loss of self processing related to a trait-based conception of the self.

One possible interpretation of our results is that parts of the dmPFC encode information about not just an awareness of the self (with regard to both traits and states) but perhaps a sense of embodiment of the self. The embodied self can be considered as a zero-sum entity due to resource limitations. A person has only one voice, one face and one body as personal traits. The more that someone portrays another person, the fewer the resources there are to devote to him/herself. One cannot speak with a British accent and Canadian accent at the same time. Therefore, acting might be akin to a deliberate process of possession, i.e. a substitution of the actor's self by the character due to their embodiment of the character. The results of the British accent condition in our experiment suggest that even when a character is not being explicitly portrayed, gestural changes through personal mimicry can be a first step towards the embodiment of a character and the retraction of the self's resources. Certain entertainers, such as ventriloquists, rapidly switch between the self and a character within the time frame of a dialogue. It would be interesting to explore what is occurring in their brains as they make these rapid but seamless transitions between self and character.

*Precuneus*. The precuneus emerged as the major area of activation increase during role change in this study (along with a weak effect seen in the pSTS/TPJ). Activation was seen here for acting when it was compared against each of the three other conditions, although the activation itself resulted from a loss of deactivation compared to those conditions. The medial parietal cortex has been well established as a component of both the mentalizing network and the default mode network (DMN) [58], and emerges as a consensus region of activation in virtually all of the meta-analyses of the ToM literature that have been published [25–31]. However, before we interpret our acting effect as a mentalizing process, it is important to note that our precuneus activation is 20–30 mm posterior to the standard peaks found for ToM tasks. A typical *y* coordinate for ToM analyses is in the −50 to −60 range, whereas our peaks were in the −70 to −80 region. Hence, this establishes a distinction between the PCC (anteriorly) and precuneus (posteriorly), where mentalizing activations tend to be overwhelmingly in the PCC. Likewise, in keeping with the role of the PCC in the DMN, the PCC was strongly deactivated in all four of the conditions in this study when contrasted with fixation (see figure 1 for the 1P results). This preferential activation of the PCC during the resting state is the typical pattern for components of the DMN [56]. However, this region did not appear in any of the high-level contrasts, suggesting that the level of deactivation of the PCC was comparable across all of the task conditions and was therefore unaffected by either perspective change or role change. Only the precuneus, but not the PCC, showed a difference when acting was compared against the other three conditions.

Regardless of whether the relative increase in activation for the precuneus for acting was due to an increase in activation or a decrease in deactivation (as was in fact the case), the question we have to address is what processes activate the precuneus. We would like to consider two literatures where the precuneus is implicated. One is attention. The precuneus is a component of the dorsal attentional network of the brain, a network that is involved in functions such as attentional orienting, episodic retrieval and mental imagery [70–72]. It is telling to point out that acting theorists for over a century have talked about the ‘split consciousness' involved in the process of acting [4,8,10]. The actor has to be himself and someone else at the same time, and this could lead to a splitting of attentional resources devoted to the focalization of attention and consciousness. This is not simply the ‘divided attention' of multi-tasking procedures, but a fundamental split of resources devoted to a maintenance of one's identity as a conscious self. According to this interpretation, activation of the precuneus would represent a dispersion of self-related attentional resources, whereas deactivation would represent a focalization or internalization of such resources. If so, then deactivation should occur during situations like mindfulness meditation, where self-related attentional resources are focalized. Garrison *et al*. [69] conducted a study in which a combined group of experienced meditators and non-meditators performed three types of mindfulness meditation in the MRI scanner (i.e. concentration, loving kindness and choiceless answers). The baseline condition was rest. The results showed that the precuneus (in addition to the PCC) was strongly deactivated during meditation, in the same region as our Fic1P versus 1P contrast. In fact, deactivations in the posterior precuneus have been reported in a number of studies of meditation [73–75]. The question for our purposes is why the precuneus effect was mainly seen for acting and not for the 3P and accent conditions. All we can say is that neither gestural modification in the form of a foreign accent nor other-orientation in the form of 3P mentalizing had an influence on this neural mechanism, whereas the explicit psychological process of role change through character portrayal did, perhaps resulting in the double consciousness that acting theorists talk about. Again, acting was the only condition in which self-identity was explicitly split during the task. Further research will be required to explore these findings.

### 4.3. Impersonation

In an interesting study of vocal impressions, McGettigan *et al*. [76] had participants perform the opening lines of familiar nursery rhymes either (i) in their normal voice, (ii) while impersonating other individuals (such as celebrities or family members) or (iii) while putting on a foreign accent. While the study was more oriented towards sensorimotor aspects of vocal performance than towards role playing or acting, the study is one of the few to address the issue of impersonation. Their impersonation versus accent contrast is in some respects quite similar to our acting versus accent contrast, although their use of familiar texts creates a difference from our improvisational question-answering task. In other words, their task is closer to an act of pure mimicry than an attempt to portray a complex character the way our actors did. The contrast of impersonation versus accent revealed activations in the pSTS, anterior STS, superior temporal gyrus and PCC. The only point of commonality with our results is their activation in the left pSTS at MNI coordinate −45, −60, 15, compared to our activation in the right TPJ for Fic1P versus Accent at Talairach coordinate 42, −61, 22 (table 2). The authors argued that activations in the pSTS and temporoparietal region might reflect the fact that ‘the emulation of specific voice identities…requires accessing the semantic knowledge of individuals' [76, p. 1882]. Beyond semantics, the pSTS has a strong connection with the perception and production of emotional expression, including not only vocal prosody but also facial expression and body gesturing [77–80]. Hence, the pSTS activation for impersonation in McGettigan *et al*. [76] and for acting in our study might reflect something about resources related to expressiveness in prosodic production.

## 5. Limitations

The present study is the first of its kind, and so it will be important for other studies to replicate the findings reported here, not least given the complex patterns of activation and deactivation that were observed. While we attempted to use a uniform group of trained actors, there are many approaches to getting into character, and so other types of actors—most especially gesture-based actors—should be analysed in future studies. In addition, we used experienced amateurs rather than professionals as our participants. It would be interesting to carry out a follow-up study with professional actors, although this would undoubtedly lead to variability in the training and performance experience of the participants, compared to the more uniform population analysed in the present study. The results with our British accent condition suggest that even small gestural manipulations, such a change in the manner of speaking, can lead to neural differences similar to full-fledged character portrayal. Outside of the domain of professional acting, there has been an explosion of interest in role-playing video games [3,81–83], and neuroimaging studies have begun to look at brain activations in gamers perceiving avatars of themselves and others while in the scanner [84]. Gamers could be yet another type of non-professional population to explore in examining neural processes of role change.

Because of our desire to compare acting with self processing and ToM, we used an improvisational question-answering task during the acting condition, rather than having the actors recite rehearsed monologues. Importantly, improvisational methods are central to the training of actors, not least the actors used in the present study. The hot-seating technique that is commonly used in rehearsal for character development follows a question-and-answer format comparable to ToM studies, with the exception that the actors are expected to answer the questions in the 1P as their characters. The participants were familiar with hot-seating technique, which was used to help them develop an attachment to their characters during the workshop phase of this study (see Methods). They were therefore familiar and comfortable with responding to hypothetical questions while in character as Romeo or Juliet. However, we cannot rule out the possibility that some of the results may be due to the fact that the actors were more personally familiar with their answers in the 1P and 3P conditions, whereas they had to ‘make up' answers in the Fic1P condition. A future study could look at the production of rehearsed dramatic monologues compared with the production of pre-learned passages generated about self-experiences (i.e. rehearsed self-monologues). Overall, future studies need to explore (i) various types of participant populations (professional actors of different training backgrounds, gamers, ventriloquists, etc.) and (ii) different types of acting tasks (rehearsed versus improvised).

## 6. Conclusion

We conducted the first neuroimaging study of dramatic role playing, with the aim of exploring how acting differs from both self processing and ToM. We did this using a group of university-trained dramatic actors having a similar type of training and a comparable degree of performance experience. The participants' ‘method acting’ approach to getting into character was ideally suited to the constraints imposed by fMRI experiments on body gesture and facial expression. In order to equate the scanner tasks across conditions, we used a question-answering task, rather than the recitation of dramatic monologues, although the latter was used by participants in the scanner as a means of getting into character immediately before the acting scan. The results showed that, compared with self processing and ToM, acting modulated processes related to attention, perspective taking and embodiment. Regarding perspective change, the contrast of acting versus self showed bilateral deactivations in the vmPFC and ventral dmPFC that were also present in the 3P versus self contrast, most probably reflecting the fact that our question-answering task required participants to tap into 3P knowledge about Romeo or Juliet while they were answering questions in character. Regarding role change, acting produced deactivations in several regions of the dmPFC and SFG dorsoventrally that resulted from a reduction in activation compared to 1P, hence a reduction of self-related resources. We argued that this might result from a reduction of trait resources related to the present self and self-embodiment. In addition, acting produced an activation in the posterior part of the precuneus that resulted from a loss of deactivation compared to the other conditions. This was the most specific effect for role change. We argued that the loss of deactivation in the precuneus for acting might represent a departure from a unified and focalized sense of attention and consciousness, towards the dual consciousness that typically characterizes dramatic acting, most especially mentalistic acting. The most surprising finding of the study was that a gestural change to one's accent while still maintaining the self-identity led to a qualitative pattern of deactivations similar to that for acting, suggesting that changes in embodiment can lead to neural changes in networks associated with perspective taking and role change. The results of this study provide a first step towards establishing a cognitive neuroscience of acting and role playing, one that considers the full gamut of processes from everyday role playing to dramatic acting [53].

## Supplementary Material

Talairach coordinates of the activations for the four tasks vs. fixation

## Supplementary Material

Talairach coordinates of the activations for four of the high-level contrasts.

## Supplementary Material

Talairach coordinates of the deactivations for the five high-level contrasts.

## Data accessibility

The datasets supporting this article have been uploaded to the Dryad Digital Repository: http://dx.doi.org/10.5061/dryad.62d6b1h [85].
